# Climate legacies drive global soil carbon stocks in terrestrial ecosystems

**DOI:** 10.1126/sciadv.1602008

**Published:** 2017-04-12

**Authors:** Manuel Delgado-Baquerizo, David J. Eldridge, Fernando T. Maestre, Senani B. Karunaratne, Pankaj Trivedi, Peter B. Reich, Brajesh K. Singh

**Affiliations:** 1 Hawkesbury Institute for the Environment, University of Western Sydney, Building L9, Locked Bag 1797, Penrith South, New South Wales 2751, Australia.; 2 Cooperative Institute for Research in Environmental Sciences, University of Colorado, Boulder, CO 80309, USA.; 3 Centre for Ecosystem Science, School of Biological, Earth and Environmental Sciences, University of New South Wales, Sydney, New South Wales 2052, Australia.; 4 Departamento de Biología y Geología, Física y Química Inorgánica, Escuela Superior de Ciencias Experimentales y Tecnología, Universidad Rey Juan Carlos, Calle Tulipán Sin Número, Móstoles 28933, Spain.; 5 Department of Bioagricultural Sciences and Pest Management, Colorado State University, Fort Collins, CO 80523, USA.; 6 Department of Forest Resources, University of Minnesota, St. Paul, MN 55108, USA.; 7 Global Centre for Land Based Innovation, University of Western Sydney, Penrith South, New South Wales 2751, Australia.

**Keywords:** Soil Carbon, Soil fertility, Climate Change, Last Glacial Maximum, Mid-Holocene, Croplands, Global scale

## Abstract

Climatic conditions shift gradually over millennia, altering the rates at which carbon (C) is fixed from the atmosphere and stored in the soil. However, legacy impacts of past climates on current soil C stocks are poorly understood. We used data from more than 5000 terrestrial sites from three global and regional data sets to identify the relative importance of current and past (Last Glacial Maximum and mid-Holocene) climatic conditions in regulating soil C stocks in natural and agricultural areas. Paleoclimate always explained a greater amount of the variance in soil C stocks than current climate at regional and global scales. Our results indicate that climatic legacies help determine global soil C stocks in terrestrial ecosystems where agriculture is highly dependent on current climatic conditions. Our findings emphasize the importance of considering how climate legacies influence soil C content, allowing us to improve quantitative predictions of global C stocks under different climatic scenarios.

## Teaser

Our findings indicate the importance of paleoclimatic information to improve quantitative predictions of global soil C stocks.

## INTRODUCTION

Soils store three times more carbon (C) than either the atmosphere or terrestrial vegetation (*1*). Moreover, soil C storage is one of the most important ecosystem processes for humans because it plays critical roles in supporting key ecosystem services such as climate regulation, soil fertility, and fiber and food production (*2*, *3*). Short-term climate legacies (that is, days to decades) have been reported to strongly influence key ecosystem processes such as plant productivity and litter decomposition in terrestrial systems (*4*–*6*). However, much less is known about the extent to which long-term climate legacies (that is, centuries to millennia) control such processes. Projections into the future are conditional upon the past, but key ecosystem and biogeochemical variables, such as soil C, strongly reflect site history across centennial to millennial time scales (*1*). Thus, quantifying the influence of long-term climate legacies on soil C stocks can be of paramount importance to better understand soil C cycle–climate change feedbacks and to improve ecosystem and earth system simulation models, which are primary tools for predicting climate change impacts on soil C at the global scale (*7*). Although a theoretical framework based on ecological principles is emerging to explain long- versus short-term legacy impacts on ecosystem functions, such as plant productivity (*5*), we still lack empirical evidence to support this framework.

**Fig. 1:**
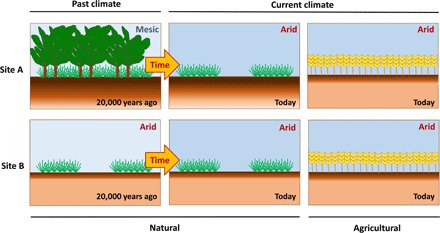
A theoretical framework explaining the effects of climatic legacies on soil C stocks in natural and agricultural areas. Higher color intensity in soil represents more soil carbon. In the example, a grassland under a current dry climate, which was previously a forest ecosystem (site A) and developed under a wetter paleoclimate now, has a greater amount of soil C than expected based on its current climate or compared to a contemporary arid grassland subjected to arid paleoclimate (site B). Shifts in land use from natural systems to agriculture have been shown to markedly reduce the amount of soil C as a result of rapid C degradation and soil erosion linked to land clearing and cultivation.

One of the most significant developments in the field of soil sciences in recent years involves the increased recognition that most soils are polygenetic, that is, archival products of pedogenic processes that vary widely over time (*8*). For example, we know from chronosequences that C accumulates in the soil over thousands of years during pedogenesis (at least as long as 10,000 years) (*8*–*12*), wherein the persistence of soil C is driven largely by abiotic and biotic factors (*9*, *12*). However, the climate of a particular area changes over decades and centuries, resulting in large-scale biome migrations (*5*). Variations in vegetation type and plant productivity, both of which are linked to shifting climate and biome migrations, regulate the rates at which C is fixed from the atmosphere via photosynthesis and stored in the soil (*13*, *14*). The long-term climate history of a region, rather than the current climatic conditions, could, in theory, be a better proxy of the amount of C that has been stored in a particular terrestrial ecosystem over many centuries. For example, a grassland under a current dry climate, which was previously a forest ecosystem under a wetter paleoclimate, may now have a greater amount of soil C than expected based on its current climate (Fig. 1) (*15*, *16*). Despite the importance of soil C for human well-being, we lack empirical evidence on the relative importance of past climates in relation to current climatic conditions as drivers of global soil C stocks in terrestrial ecosystems.

Shifts in land use from natural systems to agriculture have been shown to markedly reduce the amount of soil C as a result of rapid C degradation and soil erosion linked to land clearing and cultivation (Fig. 1) (*12*, *17*, *18*). Thus, human-induced disturbances will have a greater impact on soil C stocks than those imposed only through climate legacies in natural systems, enhancing the importance of current climate in driving soil C stocks (Fig. 1). The global pressure on soils is expected to increase exponentially during this century due to the agricultural intensification needed to meet the increasing food demand to sustain a growing global population (*19*). Improving our understanding of the role of human disturbance in shifting the relative contribution of paleo- versus current climatic conditions in regulating soil C storage is critical to improve our ability to accurately predict soil C storage in terrestrial ecosystems.

We tested the following hypotheses: (i) long-term climate legacies influence contemporary global soil C stocks in terrestrial ecosystems, and (ii) human disturbance alters the relative contribution of long-term (that is, paleoclimate) compared with current climate as drivers of these stocks. To do this, we gathered data from three independent large-scale surveys: the International Soil Reference and Information Centre’s (ISRIC’s) World Soil Information Service (“Global-WoSIS” hereafter; Materials and Methods) (*20*) including 4381 sites, data from a global field survey including 224 dryland sites from all continents except Antarctica (“Global-Drylands” hereafter) (*21*), and data from a regional survey including 450 sites scattered across a 400-km^2^ area of eastern Australia (“Australia” hereafter; see Materials and Methods) (fig. S1). These data sets included information on soil carbon stocks (kg C m^−2^; Global-WoSIS and Australia) or concentrations (%; Global-Drylands) from the uppermost soil layer (top ~10 cm). These data sets include global and regional scale data comprising a wide range of paleo- and current climatic conditions. This characteristic allows sufficient climatic variation to test the relative importance of paleo- versus current climate in predicting soil C stocks. We obtained information on 19 bioclimatic variables (Table 1) from current and paleoclimates [climates experienced in mid-Holocene (about 6000 years ago) and Last Glacial Maximum (about 22,000 years ago)] using global climate models (see Materials and Methods) (*22*, *23*). The reason for including more than one paleoclimate in our analyses is that changes in soil carbon stocks occur gradually with time as a consequence of changing conditions (*8*) rather than because of the climate of a certain time period.

**Table 1:** Bioclimatic variables included in this study.

**Bioclimatic variable**	**Acronym**
Annual mean temperature	AMT
Mean diurnal range	MDR
Isothermality	ISO
Temperature seasonality	TSEA
Maximum temperature of warmest month	MAXTWM
Minimum temperature of coldest month	MINTCM
Temperature annual range	TRANGE
Mean temperature of wettest quarter	TWETQ
Mean temperature of driest quarter	TDQ
Mean temperature of warmest quarter	TWARQ
Mean temperature of coldest quarter	TCQ
Annual precipitation	AP
Precipitation of wettest month	PWETM
Precipitation of driest month	PDM
Precipitation seasonality	PSEA
Precipitation of wettest quarter	PWETQ
Precipitation of driest quarter	PDQ
Precipitation of warmest quarter	PWARQ
Precipitation of coldest quarter	PCQ

## RESULTS AND DISCUSSION

**Fig. 2:**
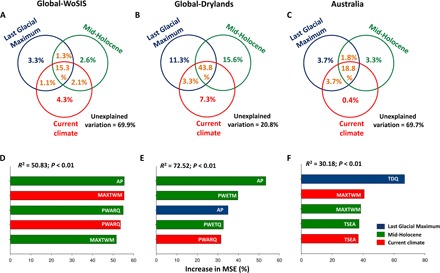
Relative contribution of paleo- (mid-Holocene and Last Glacial Maximum) and current climate as drivers of soil carbon stocks. Results from variation partitioning modeling aiming to identity the percentage of variance of soil carbon explained by past and current climate variables for the Global-WoSIS (**A**), Global-Drylands (**B**), and Australia (**C**) data sets are shown. Shared effects of these variable groups are indicated by the overlap of circles. (**D** to **F**) Results from random forest analyses aiming to identify the top five significant (*P* < 0.05) bioclimatic variables regulating soil carbon for the three data sets used. Increase in the percentage of MSE is equal to the increase in the mean square error. Acronyms are available in Table 1.

We first used variation partitioning modeling (*24*) to quantify the relative contribution of current versus mid-Holocene and Last Glacial Maximum climates in controlling contemporary soil C stocks (top ~10 cm; Materials and Methods). Variation partitioning is a method specifically recommended to deal with multicollinearity because it partitions the variance in a given response variable that is attributed to a particular group of predictors from that variance shared among all predictors (*24*). Our models explained 30.1, 79.2, and 30.3% of the variance in soil C for the Global-WoSIS, Global-Drylands, and Australia data sets, respectively (Fig. 2). They indicate that paleoclimate (mid-Holocene and Last Glacial Maximum combined) explained a greater unique amount of the variance in soil C stocks than current climate in terrestrial ecosystems at regional and global scales (5.9 versus 4.3%, 26.9 versus 7.3%, and 7.0 versus 0.5% of the variance of soil carbon in the Global-WoSIS, Global-Drylands, and Australia data sets, respectively; Fig. 2). Bioclimatic variables from the different climatic periods evaluated were highly correlated (table S1), which explains the high percentage of shared variance in predicting soil C stocks among climate periods observed (Fig. 2, A to C). Shared variation explaining C stock among different predictors (for example, current climate and mid-Holocene) cannot be attributed particularly to any of those climatic periods. Because of this, we only compared the unique portion of the variation in soil C stocks explained in a singular manner by either paleoclimate or current climate. Similar results were found after controlling for spatial influence in all cases (fig. S2) and also across bioclimatic regions (that is, tropical, temperate, continental, and arid) within the Global-WoSIS data set (fig. S3). Most importantly, these results were also maintained after explicitly accounting for other important predictors of soil C stocks such as space (latitude, longitude, and altitude), soil properties (soil pH, electrical conductivity, and sand content), and biotic factors (species richness and plant cover) simultaneously in the Global-Drylands data set, where these data were available (fig. S4). Only the Global-WoSIS data set contained soil C stock information for soil below 10-cm depth (www.isric.org/data/wosis) (*25*). Using this data set, we found a highly significant positive correlation between soil C stocks from the top 10 cm and those from all other soil depths (10 to 20, 20 to 50, 50 to 100, 0 to 20, 0 to 50, and 0 to 100 cm) (table S2). Our variation partitioning modeling provided evidence that paleoclimate still predicted a unique portion of the variance that cannot be predicted by current climate at 10 to 20, 20 to 50, 50 to 100, 0 to 20, 0 to 50, and 0 to 100 cm (figs. S5 and S6). Our findings suggest that paleoclimatic information could also be used to improve predictions of soil C stocks in the deep layers of soil (from 50 to 100 cm), where current model performance to predict soil organic C content is still largely limited (*26*).

Within the Global-WoSIS data set, the footprint of paleoclimate legacies on soil C stocks was particularly noticeable in the midlatitudes compared with the tropics (fig. S7). Here, the interaction between paleo- and current climate was a stronger driver of soil C stocks than the climate of a particular period per se (fig. S7). These differences in the role of paleoclimate in driving soil C stocks observed between midlatitude and tropical areas may be related to the extent of the last glaciation in these regions. Middle latitudes were highly affected by the last glaciations, and their climatic conditions have substantially changed over the last 20,000 years (*12*). These climatic changes likely influenced the rate of soil C fixation in these regions, and our results suggest that they have left an imprint in soil C stocks that can still be identified nowadays. Conversely, climatic conditions have been more stable in the tropics (versus middle latitudes) during the last millennia due to the lack of effects from last glaciations (*12*). Therefore, the paleoclimatic footprint is difficult to observe in these areas. Together, our findings accord with recent studies highlighting the importance of past climate as a driver of ecosystem attributes, such as litter decomposition and biodiversity in terrestrial ecosystems (*6*, *27*, *28*), and provide, to the best of our knowledge, the first empirical demonstration that climatic legacies continue to drive the magnitude of contemporary soil C stocks at regional and global scales in terrestrial ecosystems.

We used random forest modeling (*29*) to identify the main past and/or current climatic drivers of soil C stocks and structural equation modeling (SEM) (*30*) to clarify their relative influence. These techniques are specially recommended to identify the main predictors of environmental response variables (see Materials and Methods). Our random forest models explained 50.8, 72.5, and 30.2% of the variance in soil carbon for the Global-WoSIS, Global-Drylands, and Australia data sets, respectively (Fig. 2, D to F). Precipitation from paleoclimates and maximum temperature in the warmest month from current and mid-Holocene climates were the main drivers of soil C stocks at the global scale (Fig. 2, D and E; see table S3 for a complete list of the soil C stock drivers evaluated). Structural equation models suggested that, in general, paleoclimate is more important than current climate in driving soil C stocks at the global scale (fig. S8, A and B). Precipitation variables were positively related to soil C content in the two global data sets studied (table S4). Precipitation is a key driver of plant growth and litter decomposition in terrestrial ecosystems and ultimately regulates the rates at which C is fixed from the atmosphere and stored in the soil (*31*, *32*). As C accumulates in the soil over millennia in natural systems (*8*–*10*), the current soil C content reflects the climatic conditions experienced over this period. Conversely, temperature in the warmest month was negatively related to soil C content in both global data sets (table S4). Rising temperatures under climate change are predicted to promote soil C losses via soil respiration (*12*). Our study provides evidence that millennial precipitation and temperature legacies still influence soil C stocks today. Temperature in the driest quarter from the Last Glacial Maximum (positively related to soil C stocks) was the main driver of variations in soil C stocks at the regional scale in Australia (tables S4 and S5, Fig. 2F, and fig. S8C). Our random forest and SEM results suggest that paleoclimatic legacies linked to temperature regulated soil C stocks observed in eastern Australia, even within the limited precipitation range (280 to 648 mm) found in this data set (Fig. 2F and fig. S8C). Together, our findings highlight the importance of considering climate legacies on soil C stocks to improve ecosystem and earth system and global change simulation models. Implications of our results can be considered to be minimal for global drylands because of their relatively low capacity to store C (*33*). However, drylands occupy ~45% of Earth’s land mass (*34*) and regulate the global C budget. Global drylands comprise 27% (431 GT of organic C) of the global soil organic C reserves (*35*). Thus, our findings further emphasize the need to consider climatic legacies when estimating soil C stocks in global drylands. This is critical for achieving global sustainability because drylands support 38% of global human populations that rely heavily on ecosystem services linked to soil C stocks, such as forage and food production (*36*).

**Fig. 3:**
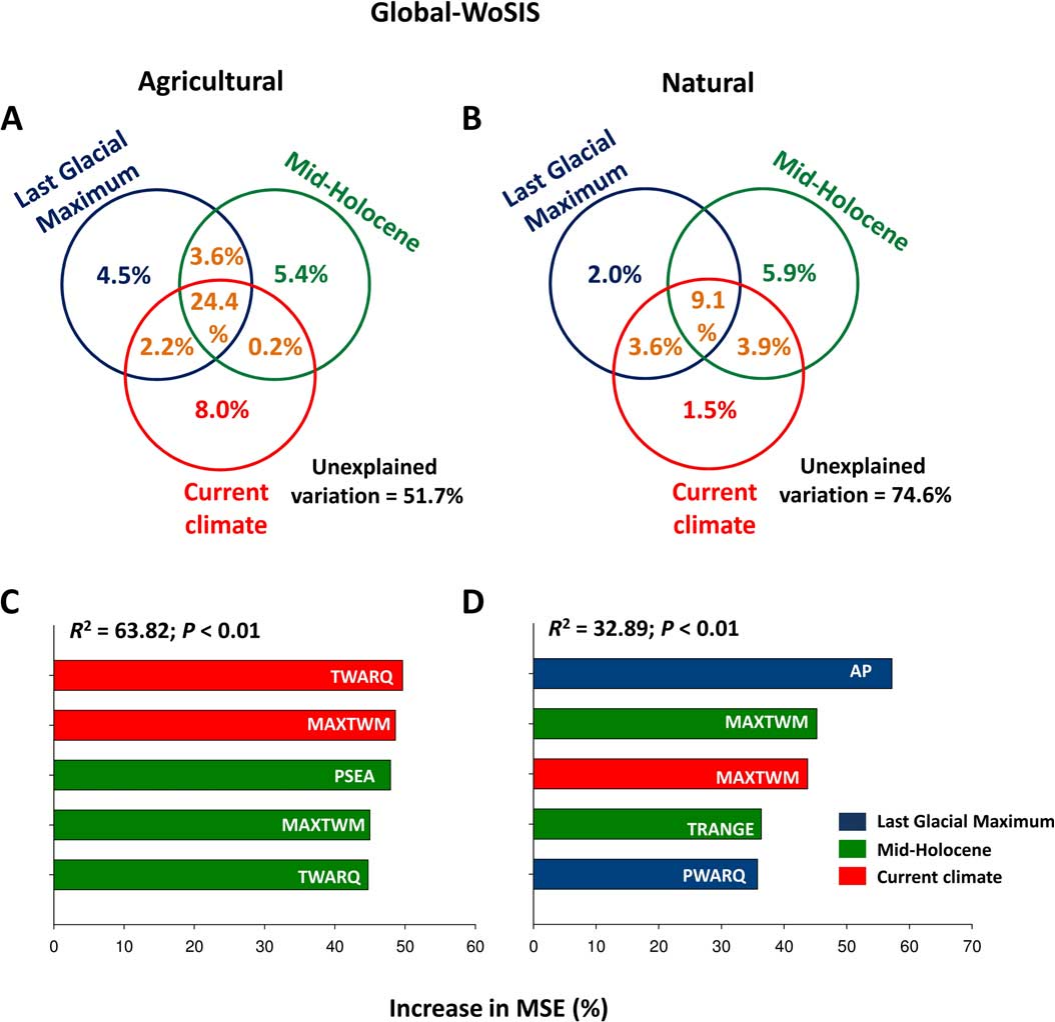
Relative contribution of paleo- (mid-Holocene and Last Glacial Maximum) and current climate as drivers of soil carbon in agricultural (*n* = 1167) and natural (*n* = 814) systems from the Global-WoSIS data set. (**A** and **B**) Variation partitioning modeling aiming to identity the percentage of variance of soil carbon explained by past and current climate variables for the identified agricultural and natural systems from the Global-WoSIS. Shared effects of these variable groups are indicated by the overlap of circles. (**C** and **D**) Results from the random forest analyses aiming to identify the top five bioclimatic variables regulating soil carbon for the three data sets used. Increase in the percentage of MSE is equal to the increase in the mean square error. Acronyms are available in Table 1.

**Fig. 4:**
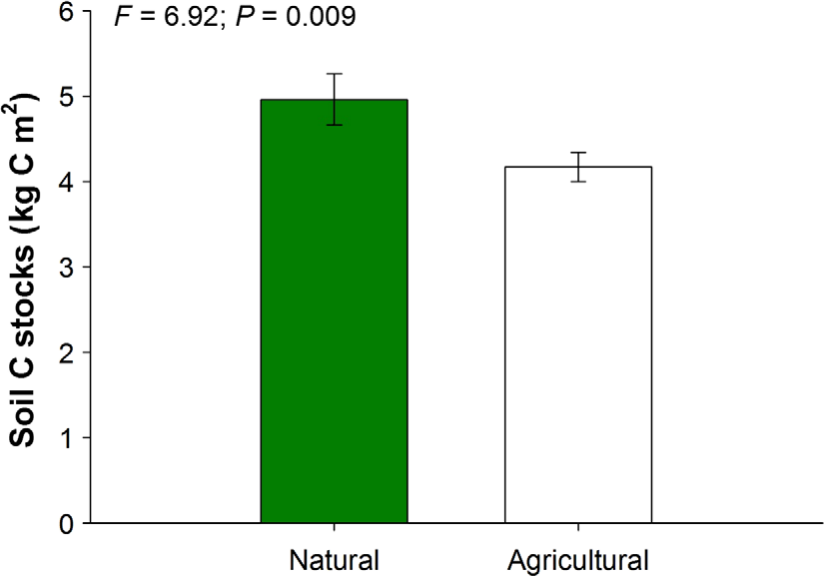
Soil carbon stocks for agricultural (*n* = 814) and natural (*n* = 1167) ecosystems from the Global-WoSIS data set. Analyses of variance (ANOVAs) were used to test for differences between natural and agricultural systems.

To test our second hypothesis (that is, soil disturbance from agriculture alters the relative contribution of paleo- compared with current climate in controlling soil C stocks), we analyzed a subset of data from the Global-WoSIS where we were able to partition sites between natural and agricultural systems (see Materials and Methods). We restricted our analyses to the Global-WoSIS data set because all samples from the Global-Drylands and Australia data sets belong to natural or seminatural ecosystems. Current climate showed a larger relative contribution to the prediction of soil C stocks for agricultural (8.0%) than natural (1.5%) systems (Fig. 3). Strikingly, in both cases, paleoclimate (mid-Holocene and Last Glacial Maximum) was still the best predictor of soil C stocks in agricultural (9.9%) and natural (7.9%) areas. Similar results were found after accounting for spatial influence in our models (fig. S9). However, paleoclimate explained five times more variance in soil C than current climate in natural compared to agricultural systems (Fig. 3 and figs. S5 and S6). These results support the hypothesis that the predictive power of current climate on soil C stocks increases with disturbances associated with agricultural practices. Reductions in soil C linked to agricultural intensification reported here (Fig. 4) and elsewhere (*12*) may promote the dependency of C stocks on current climatic conditions from a particular area after human disturbances. Although past precipitation and temperature regimes continue to be the main drivers of soil C at a global scale for natural systems (fig. S8E), current temperature (fig. S8D) and past precipitation regimes are major drivers of soil C stocks in agricultural systems (fig. S8D). These results further suggest that the greater contribution of current climate in agricultural than in natural systems may be driven more by temperature than precipitation. This is not fully unexpected because irrigation has removed the dependency of many agricultural regions on natural precipitation (*37*).

## CONCLUSIONS

Together, our findings suggest that paleoclimatic legacies should be considered in predictions of global soil C stocks in agricultural and natural terrestrial systems. Including paleoclimatic conditions in contemporary climate models will help us improve our capacity to predict soil C stocks under changing environments over the next century. This knowledge will protect us against unreasonable expectations about the likely magnitudes of increase in C stocks with short-term management practices such as minimum tillage or low-intensity grazing. Ultimately, we will be in a better position to predict how past climates are shaping our current lives and how they affect the ability of terrestrial ecosystems to provide a range of essential services, including nutrient cycling, soil carbon storage, and fiber and food production. Our findings further emphasize the importance of considering climate legacies when assessing soil C stocks to improve ecosystem and earth system simulation models, which are primary tools for predicting climate change impacts on the global C cycle.

## MATERIALS AND METHODS

### Study sites and data collection

#### Global-WoSIS

We used available online data from the ISRIC’s WoSIS (www.isric.org/data/wosis) (*20*). Here, we used 4381 sites from the Global-WoSIS, including original information on soil carbon content and bulk density across different soil horizons, which allowed us to calculate soil C stocks. We extracted 0 to 10 cm of soil C stock data (kg C m^−2^) after fitting an equal area quadratic spline (*38*). We adopted a λ value of 0.01 in fitting equal area quadratic splines for each considered soil pedon, and splines were fitted using version 2 of “Spline Tool for estimating soil attributes at standard depths” developed by CSIRO (Australian Soil Resource Information System 2011, Canberra, Australia) (www.asris.csiro.au/methods.html). Similarly, we obtained information on soil C stocks at 10 to 20, 20 to 50, and 50 to 100 cm for this data set. Data on 0 to 20, 0 to 50, and 0 to 100 cm were then calculated as the sum of C stocks for the previous estimations.

We also conducted analyses by splitting our data set into agricultural and natural ecosystems. To do so, we used four independent global land cover maps to identify the most likely land uses in each of the sites from the Global-WoSIS data set: global land cover map (GLC-2000) (*39*), GLC-SHARE (Global Land Cover Network; www.glcn.org/databases/lc_glcshare_en.jsp), Globcover2009 from the European Space Agency (http://due.esrin.esa.int/page_globcover.php), and global land cover maps obtained from the moderate resolution imaging spectroradiometer aboard NASA’s Terra satellites (http://neo.sci.gsfc.nasa.gov/view.php?datasetId=MCD12C1_T1). Only sites that were identified as agricultural or natural systems by all these global maps simultaneously (45.21% of the sites) were used for analyses, comparing agricultural to natural ecosystems. Soil C stocks were log-transformed to improve normality.

#### Global-Drylands

We used available online data from the EPES-BIOCOM global dryland survey (*21*) (www.nature.com/nature/journal/v502/n7473/source_data/nature12670-f2.xls), which focuses on dryland ecosystems, defined as regions with an aridity index (precipitation/potential evapotranspiration) between 0.05 and 0.65 (*40*). This data set includes a wide variety of habitat types (grasslands, shrublands, and open woodlands) and environmental conditions (*21*). Field data were collected between 2006 and 2010 from 224 sites located in 16 countries from all continents except Antarctica (fig. S1B) according to a standardized sampling protocol (*21*). At each site, a 30-m × 30-m plot was established under the most representative vegetation. A composite sample (that is, from five soil samples; 0 to 7.5 cm in depth) was randomly taken under the canopy of the dominant perennial plant species and in open areas devoid of perennial vegetation. After field collection, soil samples were taken to the laboratory, sieved (<2 mm), and air-dried for 1 month. Soil C (percentage) was determined using the Walkley-Black chromic acid wet oxidation method (*41*). Bulk density information was not available for the Global-Drylands data set. However, in a subset of our data set where bulk density was available, soil C stocks (kg C m^−2^) were highly correlated with soil C concentration (percentage; ρ = 0.930, *P* < 0.001, *n* = 42), providing further robustness to our approach. In the Global-Drylands data set, samples were collected in open areas and under the dominant vegetation patches; thus, all the soil variables in this data set were averaged to obtain site-level estimates by using the mean values observed in bare ground and vegetated areas, weighted by their respective cover at each site (*21*). Soil C was log_10_-transformed to improve normality.

#### Australia (regional scale)

This data set includes 450 sites scattered across a 400-km^2^ area of eastern Australia (fig. S1C). Vegetation along this transect is dominated by dense woodlands to forests of blackbox (*Eucalyptus largiflorens*), white cypress pine (*Callitris glaucophylla*), and river red gum (*Eucalyptus camaldulensis*). This data set includes sites extensively used for livestock grazing, large areas dedicated for conservation (national parks and nature reserves), and smaller areas devoted to native forestry. Field data were collected in 2014. Each site comprised a 200-m-long transect running perpendicular to the nearest livestock watering point. Along this transect, we positioned five 0.25-m^2^ (0.5 m × 0.5 m) plots every 50 m. Soils (0 to 5 cm in depth) were collected from the center of each quadrat, air-dried, ground, and passed through a 2-mm sieve to remove any roots or organic debris. Total soil C was determined using high-intensity combustion (LECO CNS-2000, LECO Corporation). Bulk density was determined with a 7-cm-diameter core to 5-cm depth, and the mass was determined after drying for 24 hours at 104°C. Soil C stocks (kg C m^−2^) (0 to 5 cm) were calculated from original total soil C and bulk density data. Soil C stocks were log_10_-transformed to improve normality.

### Climate data

A total of 19 standardized climatic variables (Table 1) were obtained from all the sites surveyed from WorldClim (www.worldclim.org) (*22*, *23*). Average changes in mean annual temperature and precipitation from Last Glacial Maximum to current climate ranged from 955 to 1033 mm at 11.5° to 17.7°C, 426 to 421 mm at 11.0° to 15.5°C, and 407 to 428 mm at 12.9° to 16.6°C for the Global-WoSIS, Global-Drylands, and Australia data sets, respectively. In the case of mid-Holocene and Last Glacial Maximum climates, we used estimates provided by the Community Climate System Model (CCSM4; www.cesm.ucar.edu/models/ccsm4.0/) (*42*–*44*). We used data at a 2.5-min resolution (~4.5 km at equator) because this is the highest resolution available for the Last Glacial Maximum period. Bioclimatic data are also available for this resolution for current and mid-Holocene climates, allowing for the direct comparison among bioclimatic data at different periods. Climatic data are also available at the 30-s resolution for the current and mid-Holocene climates, which allowed us to compare the 2.5-min and 30-s resolution data for these two periods. Values calculated using a resolution of 2.5 min were identical to those calculated using a resolution of 30 s in all cases (Pearson’s *r* > 0.99; *P* < 0.001).

### Statistical modeling

#### Variation partitioning modeling

The main goal of this analysis was to quantify the relative importance of bioclimatic variables at different periods (Table 1) as predictors of soil C stocks. In particular, this analysis provides insights into whether climatic variables from current, mid-Holocene, and Last Glacial Maximum periods can explain a unique portion of the variance that is not explained by climate in other periods (*24*, *45*). Analyses were done using C stocks (kg C m^−2^) for the Global-WoSIS and Australia data sets and C concentration (percentage) for the Global-Drylands data set; bulk density information was not available. Analyses in this section were repeated using the residuals from a multilinear regression between spatial variables (latitude and longitude) and soil C stocks as a response variable (that is, residuals of soil C stocks). The main goal of these analyses was to reduce the noise derived from spatial variables on soil C stocks because the residuals from these multilinear regressions are not influenced by either latitude or longitude. In all cases, variation partitioning analyses were conducted with the R package “vegan” (*46*). Because similar results were found across different soil depths for the Global-WoSIS data set (where this information was available) and for consistency with the other data sets included in this study, we conducted the rest of the statistical analyses using the top ~10-cm information.

#### Random forest modeling

We conducted a classification random forest analysis (*29*) to identify the main bioclimatic predictors of soil C stocks. Contrary to the variation partitioning model described above, random forest analysis allowed us to identify the most important drivers of soil C among 19 bioclimatic variables from the different climatic periods studied (Table 1). This technique is a novel machine-learning algorithm that extends standard classification and regression tree (CART) methods by creating a collection of classification trees with binary divisions. Unlike traditional CART analyses, the fit of each tree is assessed using randomly selected cases (one of three of the data), which are withheld during its construction [out-of-bag (OOB) cases]. The importance of each predictor variable is determined by evaluating the decrease in prediction accuracy (that is, increase in the mean square error between observations and OOB predictions) when the data for that predictor is randomly permuted. This decrease is averaged over all trees to produce the final measure of importance. This accuracy importance measure was computed for each tree and averaged over the forest (9999 trees). Unlike multimodel inference using linear regressions or regression tree analyses, random forest analysis alleviate multicollinearity problems in multivariate analyses by building bagged tree ensembles and including a random subset of features for each tree (9999 trees). These analyses were conducted using the randomForest package (*47*) of the R statistical software, version 3.0.2 (http://cran.r-project.org/).

#### Structural equation modeling

Using random forest analysis, we identified the top five predictors of soil C for each of the data sets and periods studied. We used SEM to further clarify, using an independent analysis, the relative importance of these soil C predictors. A useful characteristic of SEM for our purposes lies on its utility for partitioning the effects that a variable may have on another and for estimating the strengths of these multiple effects. Unlike regression or ANOVA, SEM offers the ability to separate multiple pathways of influence and view them as parts of a system and thus is useful for investigating the complex relationships among predictors commonly found in natural ecosystems (*30*). Thus, we included in our SEMs the best climatic predictor, selected by random forest analyses, at the three studied periods and soil C as our response variable. We allowed climatic predictors to covariate in these models. All the SEM analyses were conducted using AMOS 20.0.

## Supplementary Material

http://advances.sciencemag.org/cgi/content/full/3/4/e1602008/DC1

## SUPPLEMENTARY MATERIALS

Supplementary material for this article is available at http://advances.sciencemag.org/cgi/content/full/3/4/e1602008/DC1 table S1. Correlation (Pearson’s) among bioclimatic variables across different time periods. table S2. Correlations (Spearman ρ) among soil C stocks estimated at 0 to 10, 10 to 20, 20 to 50, 50 to 100, 0 to 20, 0 to 50, and 0 to 100 cm of soil depth for the Global-WoSIS data set. table S3. Results from random forest analyses aiming to identify the most important bioclimatic variables regulating soil C stocks for the three data sets used. table S4. Correlations (Spearman ρ) among bioclimatic variables across different time periods (current climate, mid-Holocene, and Last Glacial Maximum) and soil C contents for the Global-WoSIS, Global-Drylands, and Australia data sets. table S5. Direct effects of current and past climate on soil C stocks and correlations among exogenous variables (that is, climate from different periods) extending results of the structural equation models shown in fig. S6. fig. S1. Location of the sites included in the Global-WoSIS (*n* = 4381), Global-Drylands (*n* = 224), and Australia (*n* = 450) data sets. fig. S2. Relative contribution of paleo- (mid-Holocene and Last Glacial Maximum) and current climate of the residuals of soil C stocks (from a multilinear regression with latitude and longitude as predictors of soil C stocks). fig. S3. Relative contribution of paleo- versus current climate in driving soil C in tropical (*n* = 1354), temperate (*n* = 1566), continental (*n* = 655), and arid (*n* = 775) ecosystems from the Global-WoSIS data set. fig. S4. Relative contribution of paleoclimate, current climate, and other factors including space (latitude, longitude, and altitude), soil properties (soil pH, electrical conductivity, and sand content), and biotic features (total plant cover and species richness) in driving soil C stocks in the Global-Drylands data set. fig. S5. Relative contribution of paleo- versus current climate in driving soil C across different soil depths: 10 to 20 cm (all sites, *n* = 4234; agricultural sites, *n* = 1134; and natural sites, *n* = 790), 20 to 50 cm (all sites, *n* = 3797; agricultural sites, *n* = 1046; and natural sites, *n* = 670), and 50 to 100 cm (all sites, *n* = 2400; agricultural sites, *n* = 610; and natural sites, *n* = 448) for all sites available and also for the identified agricultural and natural systems from the Global-WoSIS. fig. S6. Relative contribution of paleo- versus current climate in driving soil C across different soil depths: 0 to 20 cm (all sites, *n* = 4234; agricultural sites, *n* = 1134; and natural sites, *n* = 790), 0 to 50 cm (all sites, *n* = 3786; agricultural sites, *n* = 1046; and natural sites, *n* = 674), and 0 to 100 cm (all sites, *n* = 2349; agricultural sites, *n* = 604; and natural sites, *n* = 435) for all sites available and also for the identified agricultural and natural systems from the Global-WoSIS. fig. S7. Relative contribution of paleo- versus current climate in driving soil C stocks in middle latitudes (*n* = 2080) and tropics (*n* = 2301) for the Global-WoSIS data set. fig. S8. Structural equation modeling aiming to identify the relative influence of the main bioclimatic variables from current, mid-Holocene, and land maximum climate (as identified by random forest analyses) on soil C stocks. fig. S9. Relative contribution of paleo (mid-Holocene and Last Glacial Maximum) and current climate as drivers of the residuals of soil C stocks (from a multilinear regression with latitude and longitude as predictors of soil C stocks) in agricultural (*n* = 1167) and natural (*n* = 814) systems from the Global-WoSIS data set.
