# Supplementary material for: Climate legacies drive global soil carbon stocks in terrestrial ecosystems
Source: Sci Adv. 2017 Apr 12;3(4):e1602008. doi: 10.1126/sciadv.1602008 (PMC5389782; doi:10.1126/sciadv.1602008)
Supplement: http://advances.sciencemag.org/cgi/content/full/3/4/e1602008/DC1 [file supp_3_4_e1602008__index.html]

Science Advances | Science Advances

## Supplementary Materials

**This PDF file includes:**

- table S1. Correlation (Pearson’s) among bioclimatic variables across different time periods.
- table S2. Correlations (Spearman ρ) among soil C stocks estimated at 0 to 10, 10 to 20, 20 to 50, 50 to 100, 0 to 20, 0 to 50, and 0 to 100 cm of soil depth for the Global-WoSIS data set.
- table S3. Results from random forest analyses aiming to identify the most important bioclimatic variables regulating soil C stocks for the three data sets used.
- table S4. Correlations (Spearman ρ) among bioclimatic variables across different time periods (current climate, mid-Holocene, and Last Glacial Maximum) and soil C contents for the Global-WoSIS, Global-Drylands, and Australia data sets.
- table S5. Direct effects of current and past climate on soil C stocks and correlations among exogenous variables (that is, climate from different periods) extending results of the structural equation models shown in fig. S6.
- fig. S1. Location of the sites included in the Global-WoSIS (*n* = 4381), Global-Drylands (*n* = 224), and Australia (*n* = 450) data sets.
- fig. S2. Relative contribution of paleo- (mid-Holocene and Last Glacial Maximum) and current climate of the residuals of soil C stocks (from a multilinear regression with latitude and longitude as predictors of soil C stocks).
- fig. S3. Relative contribution of paleo- versus current climate in driving soil C in tropical (*n* = 1354), temperate (*n* = 1566), continental (*n* = 655), and arid (*n* = 775) ecosystems from the Global-WoSIS data set.
- fig. S4. Relative contribution of paleoclimate, current climate, and other factors including space (latitude, longitude, and altitude), soil properties (soil pH, electrical conductivity, and sand content), and biotic features (total plant cover and species richness) in driving soil C stocks in the Global-Drylands data set.
- fig. S5. Relative contribution of paleo- versus current climate in driving soil C across different soil depths: 10 to 20 cm (all sites, *n* = 4234; agricultural sites, *n* = 1134; and natural sites, *n* = 790), 20 to 50 cm (all sites, *n* = 3797; agricultural sites, *n* = 1046; and natural sites, *n* = 670), and 50 to 100 cm (all sites, *n* = 2400; agricultural sites, *n* = 610; and natural sites, *n* = 448) for all sites available and also for the identified agricultural and natural systems from the Global-WoSIS.
- fig. S6. Relative contribution of paleo- versus current climate in driving soil C across different soil depths: 0 to 20 cm (all sites, *n* = 4234; agricultural sites, *n* = 1134; and natural sites, *n* = 790), 0 to 50 cm (all sites, *n* = 3786; agricultural sites, *n* = 1046; and natural sites, *n* = 674), and 0 to 100 cm (all sites, *n* = 2349; agricultural sites, *n* = 604; and natural sites, *n* = 435) for all sites available and also for the identified agricultural and natural systems from the Global-WoSIS.
- fig. S7. Relative contribution of paleo- versus current climate in driving soil C stocks in middle latitudes (*n* = 2080) and tropics (*n* = 2301) for the Global-WoSIS data set.
- fig. S8. Structural equation modeling aiming to identify the relative influence of the main bioclimatic variables from current, mid-Holocene, and land maximum climate (as identified by random forest analyses) on soil C stocks.
- fig. S9. Relative contribution of paleo (mid-Holocene and Last Glacial Maximum) and current climate as drivers of the residuals of soil C stocks (from a multilinear regression with latitude and longitude as predictors of soil C stocks) in agricultural (*n* = 1167) and natural (*n* = 814) systems from the Global-WoSIS data set.

Download PDF

**Other Supplementary Material for this manuscript includes the following:**

- table S1 (Microsoft Excel format). Correlation (Pearson’s) among bioclimatic variables across different time periods.

**Files in this Data Supplement:**

- Adobe PDF - 1602008\_SM.pdf
