# Supplementary material for: Climate legacies drive global soil carbon stocks in terrestrial ecosystems
Source: Sci Adv. 2017 Apr 12;3(4):e1602008. doi: 10.1126/sciadv.1602008 (PMC5389782; doi:10.1126/sciadv.1602008)
Supplement: http://advances.sciencemag.org/cgi/content/full/3/4/e1602008/DC1 [file 1602008_SM.pdf]

Science Advances

AAAS

advances.sciencemag.org/cgi/content/full/3/4/e1602008/DC1

Supplementary Materials for

Climate legacies drive global soil carbon stocks in terrestrial ecosystems

Manuel Delgado-Baquerizo, David J. Eldridge, Fernando T. Maestre, Senani B. Karunaratne, Pankaj Trivedi, Peter B. Reich, Brajesh K. Singh

Published 12 April 2017, *Sci. Adv.* **3**, e1602008 (2017)
DOI: 10.1126/sciadv.1602008

The PDF file includes:

- table S1. Correlation (Pearson's) among bioclimatic variables across different time periods.
- table S2. Correlations (Spearman ρ) among soil C stocks estimated at 0 to 10, 10 to 20, 20 to 50, 50 to 100, 0 to 20, 0 to 50, and 0 to 100 cm of soil depth for the Global-WoSIS data set.
- table S3. Results from random forest analyses aiming to identify the most important bioclimatic variables regulating soil C stocks for the three data sets used.
- table S4. Correlations (Spearman ρ) among bioclimatic variables across different time periods (current climate, mid-Holocene, and Last Glacial Maximum) and soil C contents for the Global-WoSIS, Global-Drylands, and Australia data sets.
- table S5. Direct effects of current and past climate on soil C stocks and correlations among exogenous variables (that is, climate from different periods) extending results of the structural equation models shown in fig. S6.
- fig. S1. Location of the sites included in the Global-WoSIS (n = 4381), Global-Drylands (n = 224), and Australia (n = 450) data sets.
- fig. S2. Relative contribution of paleo- (mid-Holocene and Last Glacial Maximum) and current climate of the residuals of soil C stocks (from a multilinear regression with latitude and longitude as predictors of soil C stocks).
- fig. S3. Relative contribution of paleo- versus current climate in driving soil C in tropical (n = 1354), temperate (n = 1566), continental (n = 655), and arid (n = 775) ecosystems from the Global-WoSIS data set.
- fig. S4. Relative contribution of paleoclimate, current climate, and other factors including space (latitude, longitude, and altitude), soil properties (soil pH, electrical conductivity, and sand content), and biotic features (total plant cover and species richness) in driving soil C stocks in the Global-Drylands data set.

- fig. S5. Relative contribution of paleo- versus current climate in driving soil C across different soil depths: 10 to 20 cm (all sites, *n* = 4234; agricultural sites, *n* = 1134; and natural sites, *n* = 790), 20 to 50 cm (all sites, *n* = 3797; agricultural sites, *n* = 1046; and natural sites, *n* = 670), and 50 to 100 cm (all sites, *n* = 2400; agricultural sites, *n* = 610; and natural sites, *n* = 448) for all sites available and also for the identified agricultural and natural systems from the Global-WoSIS.
- fig. S6. Relative contribution of paleo- versus current climate in driving soil C across different soil depths: 0 to 20 cm (all sites, *n* = 4234; agricultural sites, *n* = 1134; and natural sites, *n* = 790), 0 to 50 cm (all sites, *n* = 3786; agricultural sites, *n* = 1046; and natural sites, *n* = 674), and 0 to 100 cm (all sites, *n* = 2349; agricultural sites, *n* = 604; and natural sites, *n* = 435) for all sites available and also for the identified agricultural and natural systems from the Global-WoSIS.
- fig. S7. Relative contribution of paleo- versus current climate in driving soil C stocks in middle latitudes (*n* = 2080) and tropics (*n* = 2301) for the Global-WoSIS data set.
- fig. S8. Structural equation modeling aiming to identify the relative influence of the main bioclimatic variables from current, mid-Holocene, and land maximum climate (as identified by random forest analyses) on soil C stocks.
- fig. S9. Relative contribution of paleo (mid-Holocene and Last Glacial Maximum) and current climate as drivers of the residuals of soil C stocks (from a multilinear regression with latitude and longitude as predictors of soil C stocks) in agricultural (*n* = 1167) and natural (*n* = 814) systems from the Global-WoSIS data set.

Other Supplementary Material for this manuscript includes the following:
(available at advances.sciencemag.org/cgi/content/full/3/4/e1602008/DC1)

- table S1 (Microsoft Excel format). Correlation (Pearson’s) among bioclimatic variables across different time periods.

SUPPLEMENTARY MATERIALS

table S1. Correlation (Pearson's) among bioclimatic variables across different time periods. Significance levels of each predictor are as follows: *P < 0.05 and **P < 0.01. CC = Current climate; Mid-H = Mid-Holocene; LGM = Last Glacial Maximum.

Table S1 is available online as a Separate Excel file under the Supporting Information for this article.

table S2. Correlations (Spearman ρ) among soil C stocks estimated at 0 to 10, 10 to 20, 20 to 50, 50 to 100, 0 to 20, 0 to 50, and 0 to 100 cm of soil depth for the Global-WoSIS data set.

Soil depth	Parameter	0-10cm	10-20cm	20-50cm	50-100cm	0-20cm	0-50cm
**10-20cm**	**ρ**	0.928					
	***P*-value**	<0.001					
	**n**	4234					
**20-50cm**	**ρ**	0.7	0.809				
	***P*-value**	<0.001	<0.001				
	**n**	3796	3786				
**50-100cm**	**ρ**	0.449	0.518	0.708			
	***P*-value**	<0.001	<0.001	<0.001			
	**n**	2400	2393	2355			
**0-20cm**	**ρ**	0.982	0.98	0.765	0.493		
	***P*-value**	<0.001	<0.001	<0.001	<0.001		
	**n**	4234	4234	3786	2393		
**0-50cm**	**ρ**	0.941	0.977	0.872	0.591	0.977	
	***P*-value**	<0.001	<0.001	<0.001	<0.001	<0.001	
	**n**	3786	3786	3786	2349	3786	
**0-100cm**	**ρ**	0.885	0.93	0.88	0.725	0.929	0.977
	***P*-value**	<0.001	<0.001	<0.001	<0.001	<0.001	<0.001
	**n**	2349	2349	2349	2349	2349	2349

table S3. Results from random forest analyses aiming to identify the most important bioclimatic variables regulating soil C stocks for the three data sets used. Increase in % of MSE = Increase in the mean square error. Acronyms of climatic variables are shown in Table 1. CC = Current climate; MH = mid-Holocene; LGM = Last Glacial Maximum

Top	Global-WoSIS		Global-Drylands		Australia	
**1**	**MH-AP**	55.43	**MH-AP**	53.40	**LGM-TDQ**	66.81
**2**	**CC-MAXTWM**	55.37	**MH-PWETM**	39.79	**CC-MAXTWM**	40.71
**3**	**MH-PWARQ**	54.93	**LGM-AP**	34.96	**MH-MAXTWM**	38.51
**4**	**CC-PWARQ**	53.58	**MH-PWETQ**	32.72	**MH-TSEA**	37.41
**5**	**MH-MAXTWM**	51.57	**CC-PWARQ**	31.54	**CC-TSEA**	36.89
**6**	**LGM-PWARQ**	51.54	**CC-MDR**	31.37	**CC-TWETQ**	35.63
**7**	**CC-TDQ**	51.21	**CC-TWARQ**	31.16	**CC-PSEA**	34.40
**8**	**CC-PCQ**	49.89	**CC-AP**	30.46	**MH-TDQ**	33.88
**9**	**CC-AP**	48.34	**LGM-TSEA**	29.03	**LGM-TRANGE**	32.36
**10**	**LGM-AP**	47.19	**MH-PSEA**	28.70	**CC-TRANGE**	32.31
**11**	**MH-TSEA**	47.04	**LGM-MDR**	28.66	**LGM-AMT**	32.07
**12**	**MH-TWETQ**	46.62	**MH-PCQ**	28.48	**MH-MDR**	32.05
**13**	**CC-MDR**	44.98	**LGM-PSEA**	28.34	**MH-PSEA**	31.12
**14**	**CC-PSEA**	44.76	**MH-MDR**	27.90	**LGM-TSEA**	30.27
**15**	**LGM-PCQ**	44.08	**LGM-AMT**	27.71	**CC-PWETQ**	30.14
**16**	**LGM-TSEA**	43.38	**MH-TWARQ**	27.36	**LGM-PWETQ**	29.54
**17**	**CC-PWETQ**	42.95	**MH-TWETQ**	27.18	**LGM-TWETQ**	29.35
**18**	**LGM-TRANGE**	42.69	**CC-MAXTWM**	27.16	**LGM-PWARQ**	28.94
**19**	**CC-TRANGE**	42.56	**LGM-TWETQ**	26.99	**CC-TDQ**	28.49
**20**	**CC-TSEA**	42.50	**LGM-TWARQ**	26.86	**CC-TWARQ**	28.38
**21**	**MH-TRANGE**	42.15	**LGM-MAXTWM**	25.44	**LGM-AP**	28.31
**22**	**LGM-MINTCM**	41.52	**MH-PWARQ**	25.32	**MH-PCQ**	28.16
**23**	**MH-TWARQ**	41.41	**LGM-PDQ**	24.93	**LGM-MAXTWM**	27.87
**24**	**MH-TDQ**	41.21	**LGM-PWARQ**	24.25	**MH-TRANGE**	27.46
**25**	**MH-PWETQ**	40.71	**MH-PDQ**	23.88	**LGM-PDQ**	27.40
**26**	**LGM-TDQ**	40.67	**CC-AMT**	23.82	**LGM-PWETM**	27.19
**27**	**CC-TWETQ**	38.89	**LGM-PWETM**	23.77	**CC-MDR**	27.13

**28**	**MH-AMT**	38.81	**MH-MAXTWM**	23.31	**LGM-MINTCM**	26.65
**29**	**LGM-MDR**	38.74	**CC-PCQ**	23.07	**MH-TWETQ**	26.17
**30**	**LGM-MAXTWM**	38.68	**LGM-PCQ**	22.99	**MH-PDM**	25.92
**31**	**CC-AMT**	38.50	**MH-AMT**	22.31	**MH-TWARQ**	25.83
**32**	**MH-PDM**	38.09	**CC-TWETQ**	22.03	**LGM-PCQ**	25.77
**33**	**MH-PCQ**	37.80	**LGM-PWETQ**	21.98	**CC-AP**	25.73
**34**	**LGM-TCQ**	37.60	**CC-PWETQ**	21.95	**CC-PCQ**	25.55
**35**	**LGM-TWETQ**	37.58	**CC-TSEA**	21.68	**CC-PWARQ**	25.36
**36**	**MH-PWETM**	37.18	**MH-TCQ**	21.14	**MH-PWETQ**	24.83
**37**	**MH-ISO**	36.98	**CC-TDQ**	21.08	**MH-AMT**	24.67
**38**	**LGM-AMT**	36.98	**LGM-TDQ**	21.02	**MH-AP**	24.50
**39**	**CC-PDQ**	36.85	**CC-PDQ**	20.79	**CC-PWETM**	23.84
**40**	**MH-PDQ**	36.11	**CC-ISO**	20.14	**LGM-PDM**	23.49
**41**	**CC-TWARQ**	36.11	**LGM-TCQ**	18.88	**CC-PDM**	23.44
**42**	**LGM-PDQ**	35.91	**MH-TDQ**	18.85	**MH-PWETM**	23.37
**43**	**CC-PDM**	35.59	**MH-PDM**	18.82	**MH-PWARQ**	23.25
**44**	**MH-PSEA**	35.46	**MH-TSEA**	18.78	**CC-PDQ**	22.38
**45**	**CC-ISO**	34.86	**CC-PWETM**	18.25	**CC-AMT**	22.27
**46**	**LGM-TWARQ**	34.86	**LGM-TRANGE**	17.96	**MH-PDQ**	21.63
**47**	**MH-MINTCM**	34.63	**CC-TCQ**	17.91	**LGM-TWARQ**	20.98
**48**	**LGM-PWETQ**	34.52	**MH-ISO**	17.87	**LGM-MDR**	20.34
**49**	**CC-MINTCM**	34.20	**LGM-ISO**	17.79	**CC-MINTCM**	19.21
**50**	**CC-TCQ**	34.04	**CC-PSEA**	17.09	**LGM-TCQ**	19.15
**51**	**MH-MDR**	32.98	**MH-MINTCM**	17.08	**LGM-PSEA**	18.46
**52**	**CC-PWETM**	32.59	**CC-PDM**	16.84	**CC-TCQ**	16.90
**53**	**MH-TCQ**	32.50	**LGM-MINTCM**	16.55	**MH-MINTCM**	16.84
**54**	**LGM-PSEA**	31.02	**CC-MINTCM**	16.20	**MH-TCQ**	16.53
**55**	**LGM-ISO**	29.57	**CC-TRANGE**	15.90	**CC-ISO**	14.51
**56**	**LGM-PWETM**	28.54	**LGM-PDM**	15.55	**MH-ISO**	14.48
**57**	**LGM-PDM**	28.50	**MH-TRANGE**	14.06	**LGM-ISO**	12.90

table S4. Correlations (Spearman ρ) among bioclimatic variables across different time periods (current climate, mid-Holocene, and Last Glacial Maximum) and soil C contents for the Global-WoSIS, Global-Drylands, and Australia data sets. Significance levels of each predictor are as follows: *$P < 0.05$ and **$P < 0.01$. CC = Current climate; MH = Mid-Holocene; LGM = Last Glacial Maximum.

		Global-WoSIS			Global-Drylands			Australia		
**Bioclimatic variable**	**Acronym**	**CC**	**MH**	**LGM**	**CC**	**MH**	**LGM**	**CC**	**MH**	**LGM**
Annual Mean Temperature	AMT	-0.225**	-0.222**	-0.219**	-0.071	-0.077	-0.049	-0.403**	-0.410**	-0.409**
Mean Diurnal Range	MDR	-0.121**	-0.089**	-0.101**	-0.293**	-0.282**	-0.370**	-0.278**	-0.130**	-0.046
Isothermality	ISO	0.042**	0.059**	0.041**	-0.187**	-0.176**	-0.016	0.122**	0.214**	0.156**
Temperature Seasonality	TSEA	-0.058**	-0.056**	-0.043**	0.061	0.076	-0.229**	-0.281**	-0.306**	-0.206**
Max Temperature of Warmest Month	MAXTWM	-0.262**	-0.270**	-0.237**	-0.235**	-0.099	-0.262**	-0.441**	-0.436**	-0.383**
Min Temperature of Coldest Month	MINTCM	-0.109**	-0.100**	-0.104**	0.02	0.022	0.080	-0.155**	-0.196**	-0.208**
Temperature Annual Range	TRANGE	-0.070**	-0.071**	-0.055**	-0.009	0.045	-0.240**	-0.369**	-0.310**	-0.167**
Mean Temperature of Wettest Quarter	TWETQ	-0.163**	-0.156**	-0.233**	-0.017	-0.042	-0.095	-0.375**	-0.378**	-0.337**
Mean Temperature of Driest Quarter	TDQ	-0.184**	-0.171**	-0.155**	0.156*	0.177**	0.033	-0.076	-0.075	0.259**
Mean Temperature of Warmest Quarter	TWARQ	-0.254**	-0.263**	-0.245**	-0.221**	-0.137*	-0.234**	-0.387**	-0.394**	-0.377**
Mean Temperature of Coldest Quarter	TCQ	-0.163**	-0.140**	-0.149**	-0.034	-0.038	0.017	-0.342**	-0.350**	-0.341**
Annual Precipitation	AP	0.216**	0.135**	0.223**	0.509**	0.559**	0.525**	0.275**	0.263**	0.250**
Precipitation of Wettest Month	PWETM	0.131**	0.041**	0.211**	0.297**	0.358**	0.382**	0.227**	0.109*	0.243**
Precipitation of Driest Month	PDM	0.208**	0.214**	0.190**	0.389**	0.399**	0.340**	0.171**	0.060	0.304**
Precipitation Seasonality	PSEA	-0.135**	-0.146**	-0.105**	-0.253**	-0.230**	-0.242**	0.082	0.057	-0.139**
Precipitation of Wettest Quarter	PWETQ	0.146**	0.053**	0.216**	0.302**	0.355**	0.376**	0.316**	0.300**	0.175**

Precipitation of Driest Quarter	PDQ	0.211**	0.224**	0.200**	0.451**	0.490**	0.425**	0.091	0.068	0.157**
Precipitation of Warmest Quarter	PWARQ	0.238**	0.236**	0.204**	0.105	0.160*	0.110	0.038	0.053	0.006
Precipitation of Coldest Quarter	PCQ	0.220**	0.194**	0.190**	0.382**	0.462**	0.305**	0.370**	0.382**	0.366**

table S5. Direct effects of current and past climate on soil C stocks and correlations among exogenous variables (that is, climate from different periods) extending results of the structural equation models shown in fig. S6.

	Variables			Estimate	P
**fig. S6a**	Carbon stocks	<---	MAXTWM-CC	-0.154	0.006
	Carbon stocks	<---	MAXTWM-MH	-0.16	0.004
	Carbon stocks	<---	AP-MH	0.149	0.001
	Carbon stocks	<---	PWARQ-MH	0.079	0.002
	Carbon stocks	<---	PWARQ-CC	-0.043	0.104
	PWARQ-MH	<-->	PWARQ-CC	0.833	0.001
	AP-MH	<-->	PWARQ-CC	0.638	0.001
	MAXTWM-MH	<-->	PWARQ-CC	-0.215	0.001
	MAXTWM-CC	<-->	PWARQ-CC	-0.183	0.001
	AP-MH	<-->	PWARQ-MH	0.754	0.001
	MAXTWM-MH	<-->	PWARQ-MH	-0.14	0.001
	MAXTWM-CC	<-->	PWARQ-MH	-0.082	0.001
	MAXTWM-MH	<-->	AP-MH	0.023	0.085
	MAXTWM-CC	<-->	AP-MH	0.104	0.001
	MAXTWM-CC	<-->	MAXTWM-MH	0.951	0.002
**fig. S6b**	Carbon stocks	<---	PWARQ-CC	-0.004	0.963
	Carbon stocks	<---	AP-MH	0.946	0.002
	Carbon stocks	<---	PWETM-MH	0.679	0.247
	Carbon stocks	<---	PWETQ-MH	-1.674	0.001
	Carbon stocks	<---	AP-LGM	0.299	0.072
	ccmidbi16	<-->	AP-LGM	0.829	0.001
	PWETM-MH	<-->	AP-LGM	0.796	0.001
	AP-MH	<-->	AP-LGM	0.944	0.001
	PWARQ-CC	<-->	AP-LGM	0.368	0.001
	PWETM-MH	<-->	PWETQ-MH	0.99	0.001
	AP-MH	<-->	PWETQ-MH	0.906	0.001
	PWARQ-CC	<-->	PWETQ-MH	0.504	0.002
	AP-MH	<-->	PWETM-MH	0.89	0.001
	PWARQ-CC	<-->	PWETM-MH	0.49	0.002
	PWARQ-CC	<-->	AP-MH	0.451	0.001
**fig. S6c**	Carbon stocks	<---	MAXTWM-CC	-0.456	0.52
	Carbon stocks	<---	TSEA-CC	1.075	0.069

	Carbon stocks	<---	MAXTWM-MH	0.127	0.855
	Carbon stocks	<---	TSEA-MH	-1.021	0.11
	Carbon stocks	<---	TDQ-LGM	0.151	0.006
	TSEA-MH	<-->	TDQ-LGM	-0.459	0.001
	MAXTWM-MH	<-->	TDQ-LGM	-0.427	0.002
	TSEA-CC	<-->	TDQ-LGM	-0.414	0.001
	MAXTWM-CC	<-->	TDQ-LGM	-0.4	0.001
	MAXTWM-MH	<-->	TSEA-MH	0.781	0.001
	TSEA-CC	<-->	TSEA-MH	0.994	0.001
	MAXTWM-CC	<-->	TSEA-MH	0.763	0.002
	TSEA-CC	<-->	MAXTWM-MH	0.731	0.001
	MAXTWM-CC	<-->	MAXTWM-MH	0.998	0.001
	MAXTWM-CC	<-->	TSEA-CC	0.714	0.001
**fig. S6d**	Carbon stocks	<---	MAXTWM-CC	2.029	0.001
	Carbon stocks	<---	MAXTWM-MH	-1.274	0.001
	Carbon stocks	<---	TWARQ-MH	1.44	0.001
	Carbon stocks	<---	TWARQ-CC	-2.418	0.001
	Carbon stocks	<---	PSEA-MH	0.101	0.019
	TWARQ-CC	<-->	PSEA-MH	0.713	0.001
	TWARQ-MH	<-->	PSEA-MH	0.552	0.001
	MAXTWM-MH	<-->	PSEA-MH	0.632	0.001
	MAXTWM-CC	<-->	PSEA-MH	0.777	0.001
	TWARQ-MH	<-->	TWARQ-CC	0.956	0.001
	MAXTWM-MH	<-->	TWARQ-CC	0.946	0.001
	MAXTWM-CC	<-->	TWARQ-CC	0.977	0.001
	MAXTWM-MH	<-->	TWARQ-MH	0.962	0.001
	MAXTWM-CC	<-->	TWARQ-MH	0.906	0.001
	MAXTWM-CC	<-->	MAXTWM-MH	0.952	0.001
**fig. S6e**	Carbon stocks	<---	MAXTWM-CC	-0.628	0.002
	Carbon stocks	<---	MAXTWM-MH	0.253	0.16
	Carbon stocks	<---	TRANGE-MH	-0.112	0.047
	Carbon stocks	<---	AP-LGM	0.292	0.001
	Carbon stocks	<---	PWARQ-LGM	-0.166	0.005
	AP-LGM	<-->	PWARQ-LGM	0.77	0.002
	TRANGE-MH	<-->	PWARQ-LGM	-0.396	0.002
	MAXTWM-MH	<-->	PWARQ-LGM	-0.029	0.366
	MAXTWM-CC	<-->	PWARQ-LGM	-0.031	0.286
	TRANGE-MH	<-->	AP-LGM	-0.58	0.002
	MAXTWM-MH	<-->	AP-LGM	0.104	0.002

MAXTWM-CC	<-->	AP-LGM	0.124	0.001
MAXTWM-MH	<-->	TRANGE-MH	-0.101	0.003
MAXTWM-CC	<-->	TRANGE-MH	-0.215	0.001
MAXTWM-CC	<-->	MAXTWM-MH	0.974	0.001

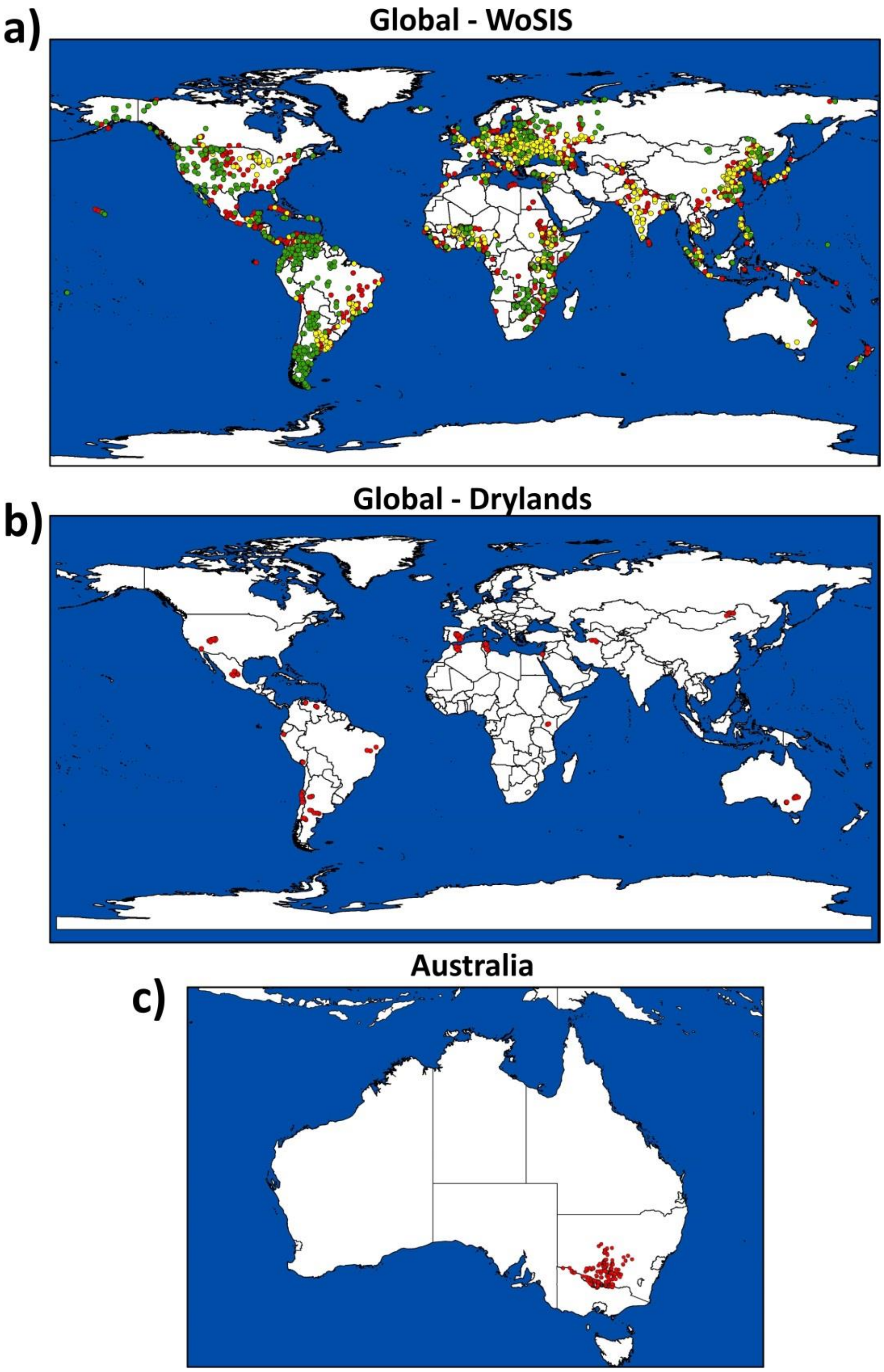

fig. S1. Location of the sites included in the Global-WoSIS (*n* = 4381), Global-Drylands (*n* = 224), and Australia (*n* = 450) data sets. In panel (**a**) green and yellow points indicate natural (n = 1167) and agricultural (n = 814) ecosystems, respectively.

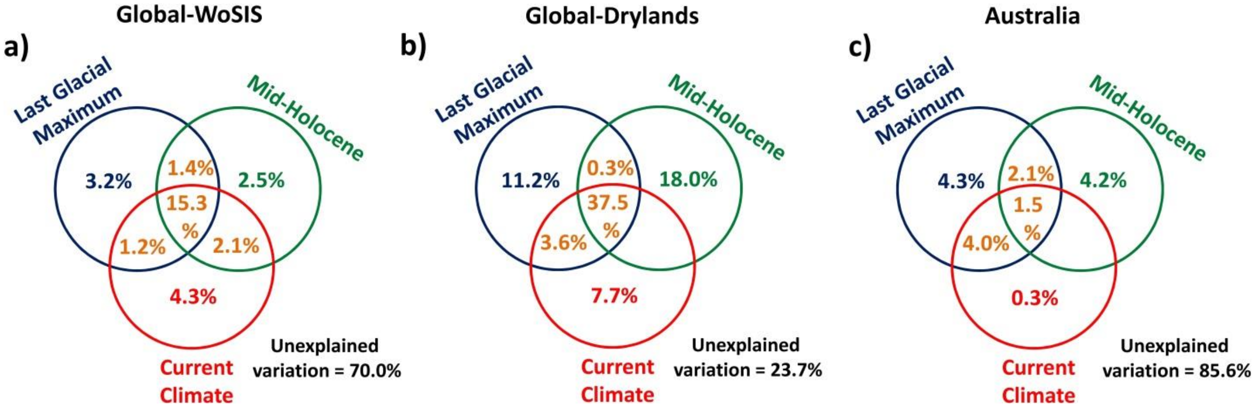

fig. S2. Relative contribution of paleo- (mid-Holocene and Last Glacial Maximum) and current climate of the residuals of soil C stocks (from a multilinear regression with latitude and longitude as predictors of soil C stocks). Variation partitioning modelling aiming to identity the % variance of residuals of soil C stock explained by past and current climate variables for the Global-WoSIS, Global-Drylands and Australia datasets. The main goal of these analyses was to reduce the noise derived from spatial variables on soil C stocks. Thus, the residuals from these multi-linear regressions were not influenced by either latitude or longitude.

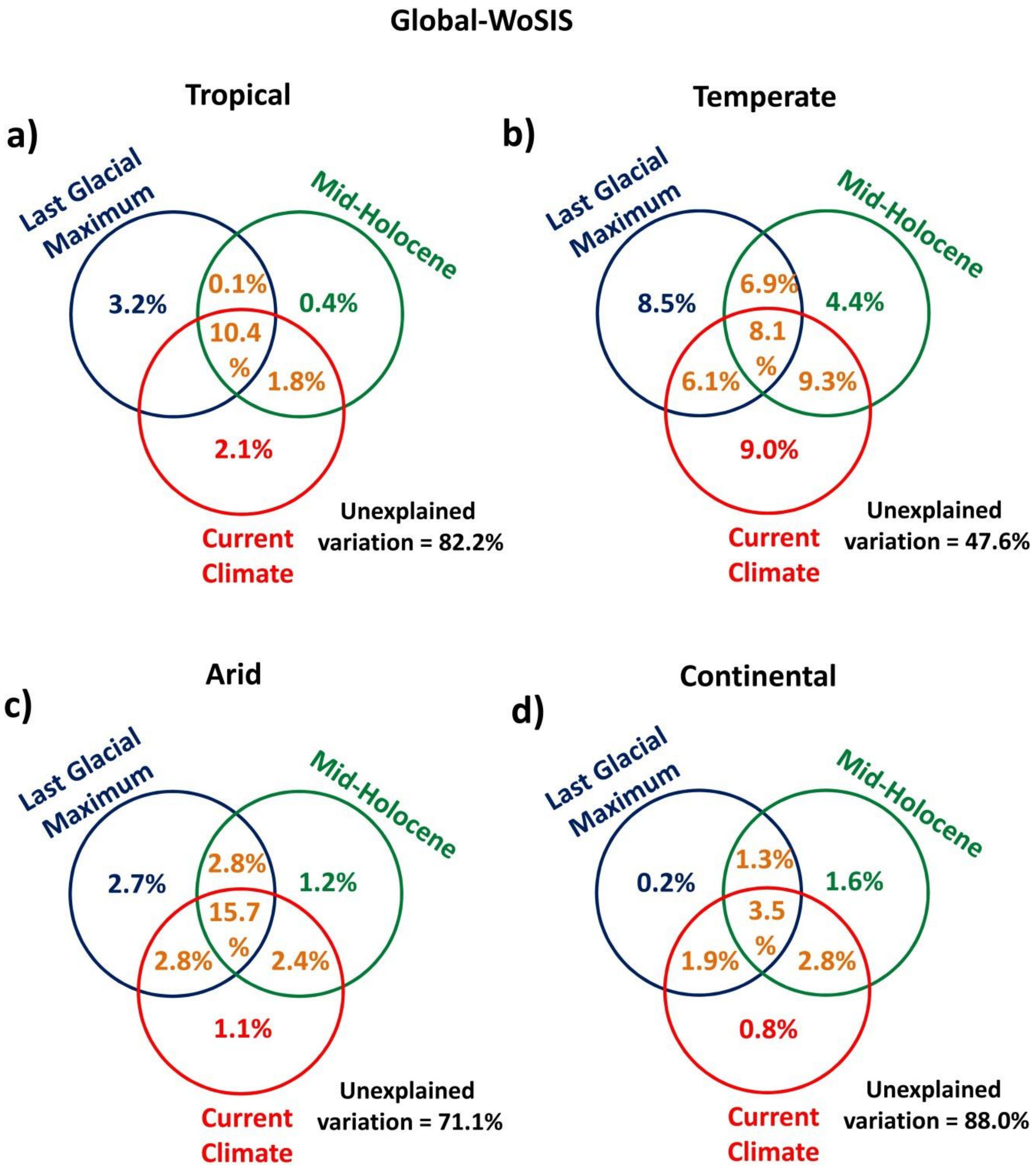

fig. S3. Relative contribution of paleo- versus current climate in driving soil C in tropical (*n* = 1354), temperate (*n* = 1566), continental (*n* = 655), and arid (*n* = 775) ecosystems from the Global-WoSIS data set. Variation partitioning modelling aiming to identity the % variance of soil C explained by bioclimatic variables from current climate, mid-Holocene and Last Glacial Maximum. Shared effects of these variable groups are indicated by the overlap of circles.

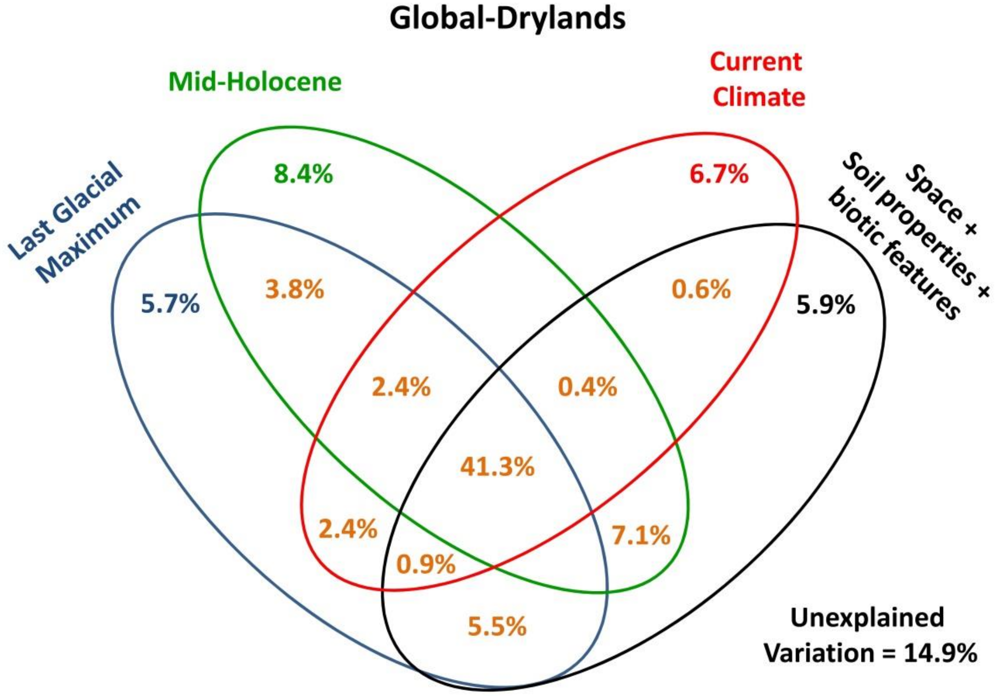

fig. S4. Relative contribution of paleoclimate, current climate, and other factors including space (latitude, longitude, and altitude), soil properties (soil pH, electrical conductivity, and sand content), and biotic features (total plant cover and species richness) in driving soil C stocks in the Global-Drylands data set. Shared effects of these variable groups are indicated by the overlap of circles.

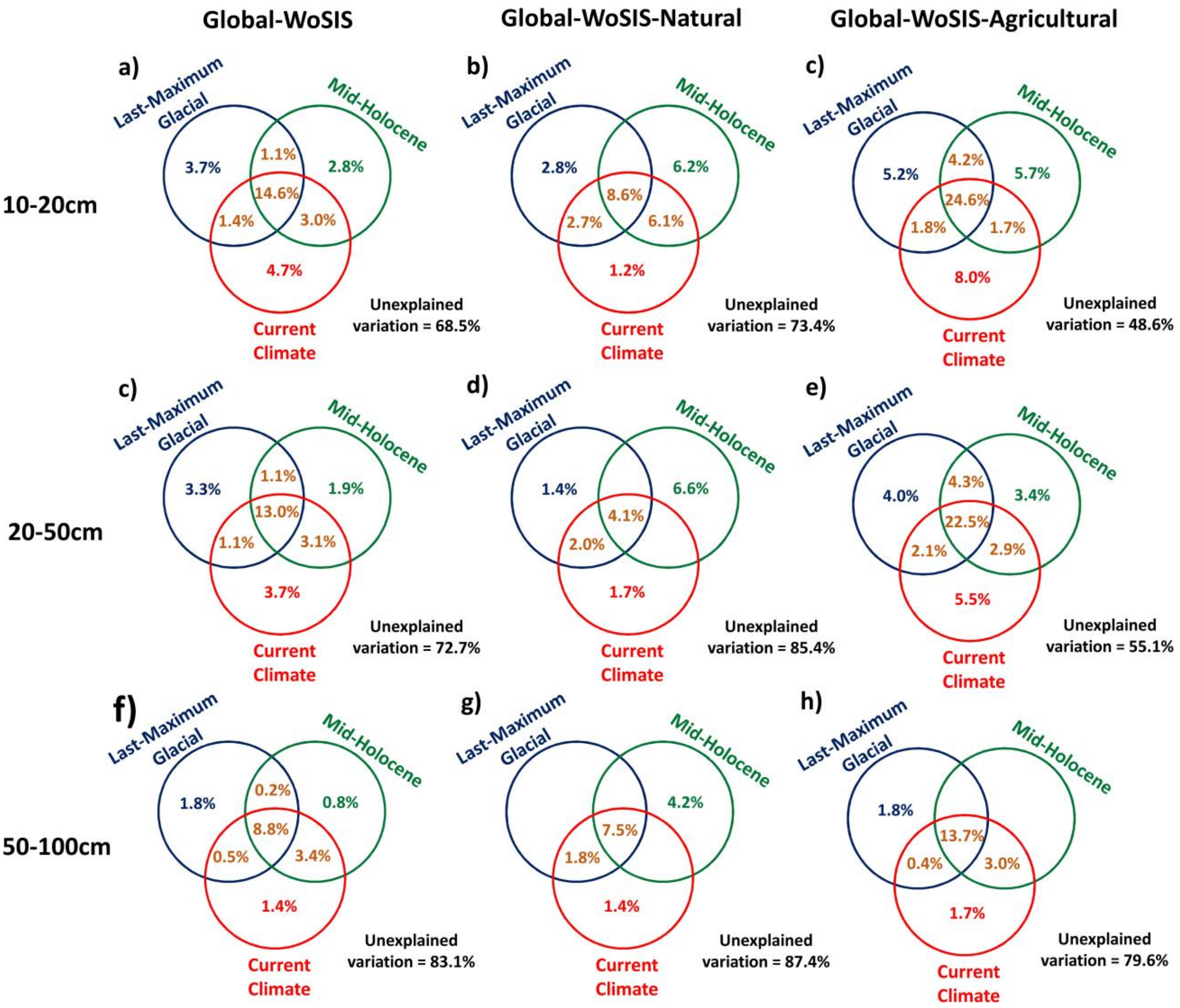

fig. S5. Relative contribution of paleo- versus current climate in driving soil C across different soil depths: 10 to 20 cm (all sites, *n* = 4234; agricultural sites, *n* = 1134; and natural sites, *n* = 790), 20 to 50 cm (all sites, *n* = 3797; agricultural sites, *n* = 1046; and natural sites, *n* = 670), and 50 to 100 cm (all sites, *n* = 2400; agricultural sites, *n* = 610; and natural sites, *n* = 448) for all sites available and also for the identified agricultural and natural systems from the Global-WoSIS. Variation partitioning modelling aiming to identity the % variance of soil C explained by bioclimatic variables from current climate, mid-Holocene and Last Glacial Maximum. Shared effects of these variable groups are indicated by the overlap of circles.

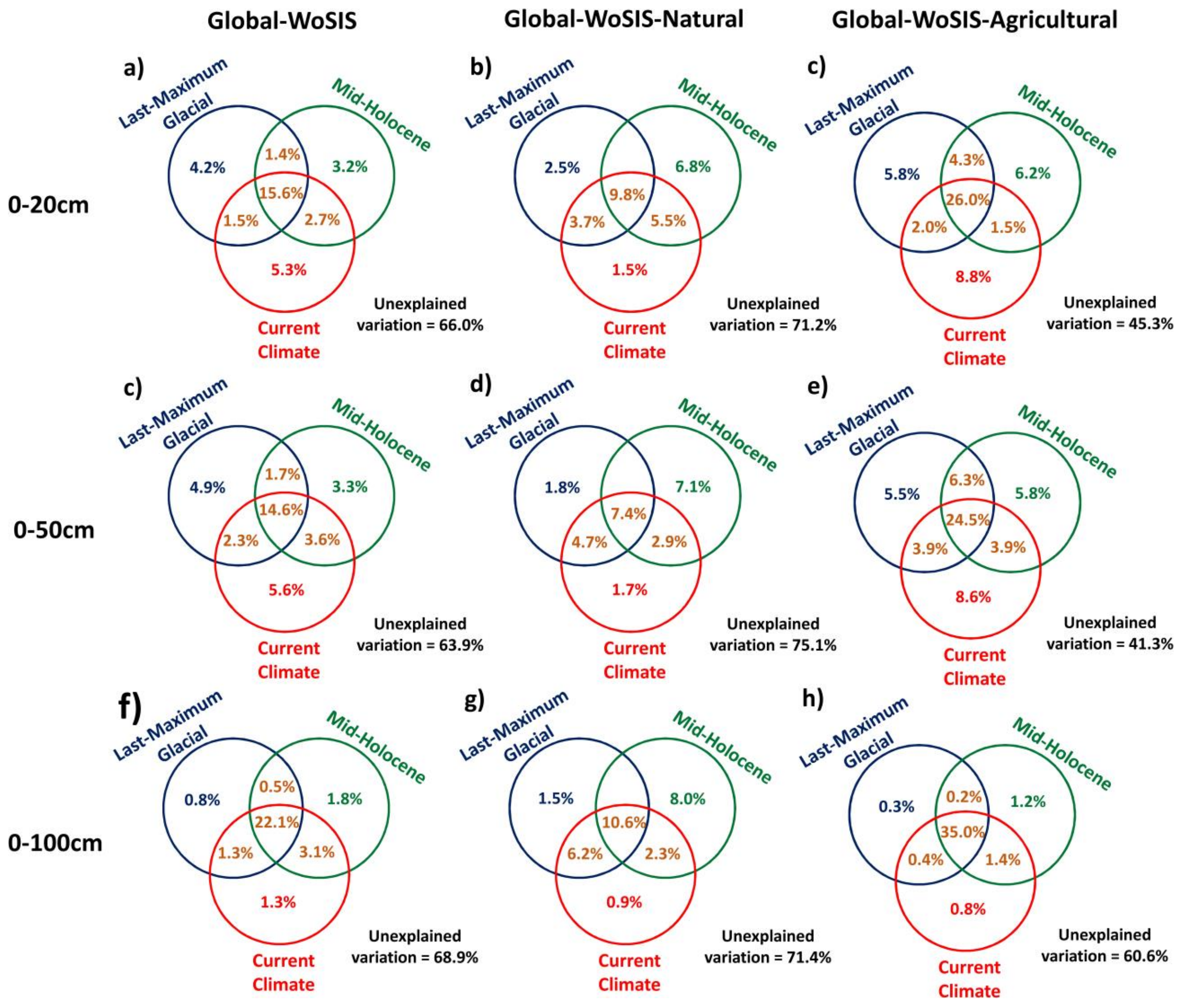

fig. S6. Relative contribution of paleo- versus current climate in driving soil C across different soil depths: 0 to 20 cm (all sites, *n* = 4234; agricultural sites, *n* = 1134; and natural sites, *n* = 790), 0 to 50 cm (all sites, *n* = 3786; agricultural sites, *n* = 1046; and natural sites, *n* = 674), and 0 to 100 cm (all sites, *n* = 2349; agricultural sites, *n* = 604; and natural sites, *n* = 435) for all sites available and also for the identified agricultural and natural systems from the Global-WoSIS. Variation partitioning modelling aiming to identity the % variance of soil C explained by bioclimatic variables from current climate, mid-Holocene and Last Glacial Maximum. Shared effects of these variable groups are indicated by the overlap of circles.

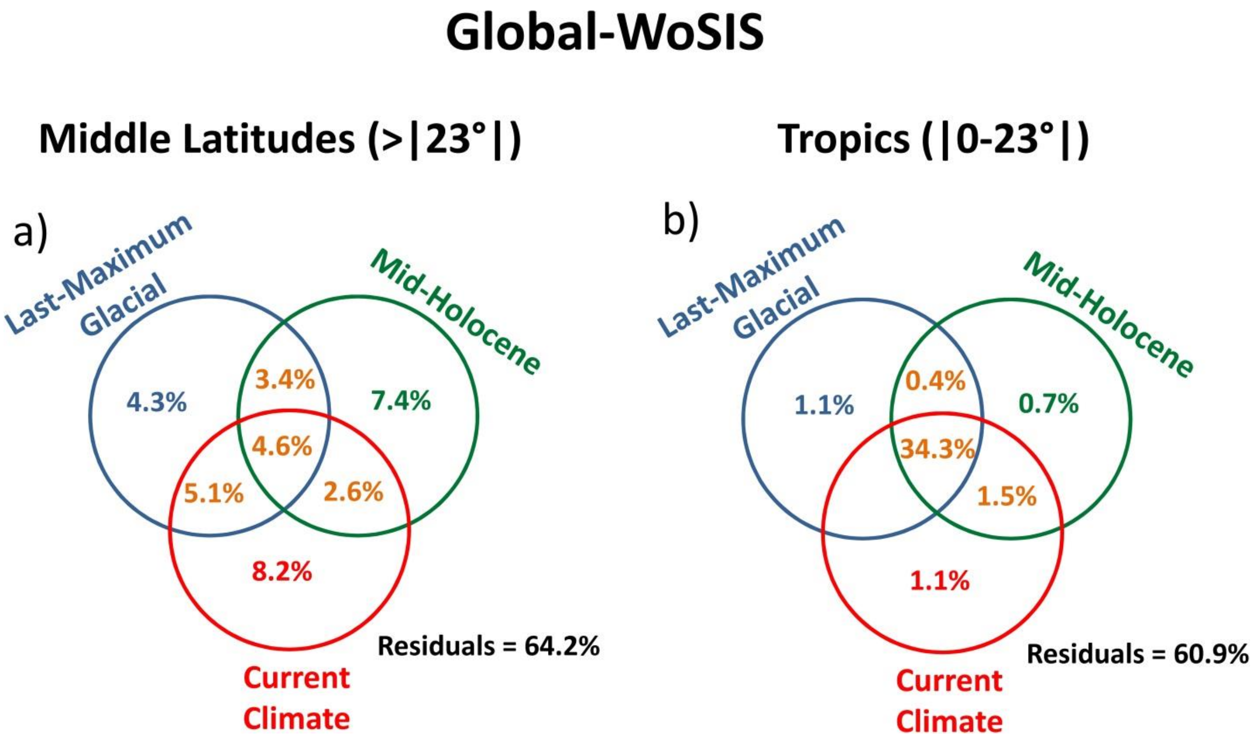

fig. S7. Relative contribution of paleo- versus current climate in driving soil C stocks in middle latitudes (*n* = 2080) and tropics (*n* = 2301) for the Global-WoSIS data set. Variation partitioning modelling aiming to identity the % variance of soil C explained by bioclimatic variables from current climate, mid-Holocene and Last Glacial Maximum. Shared effects of these variable groups are indicated by the overlap of circle.

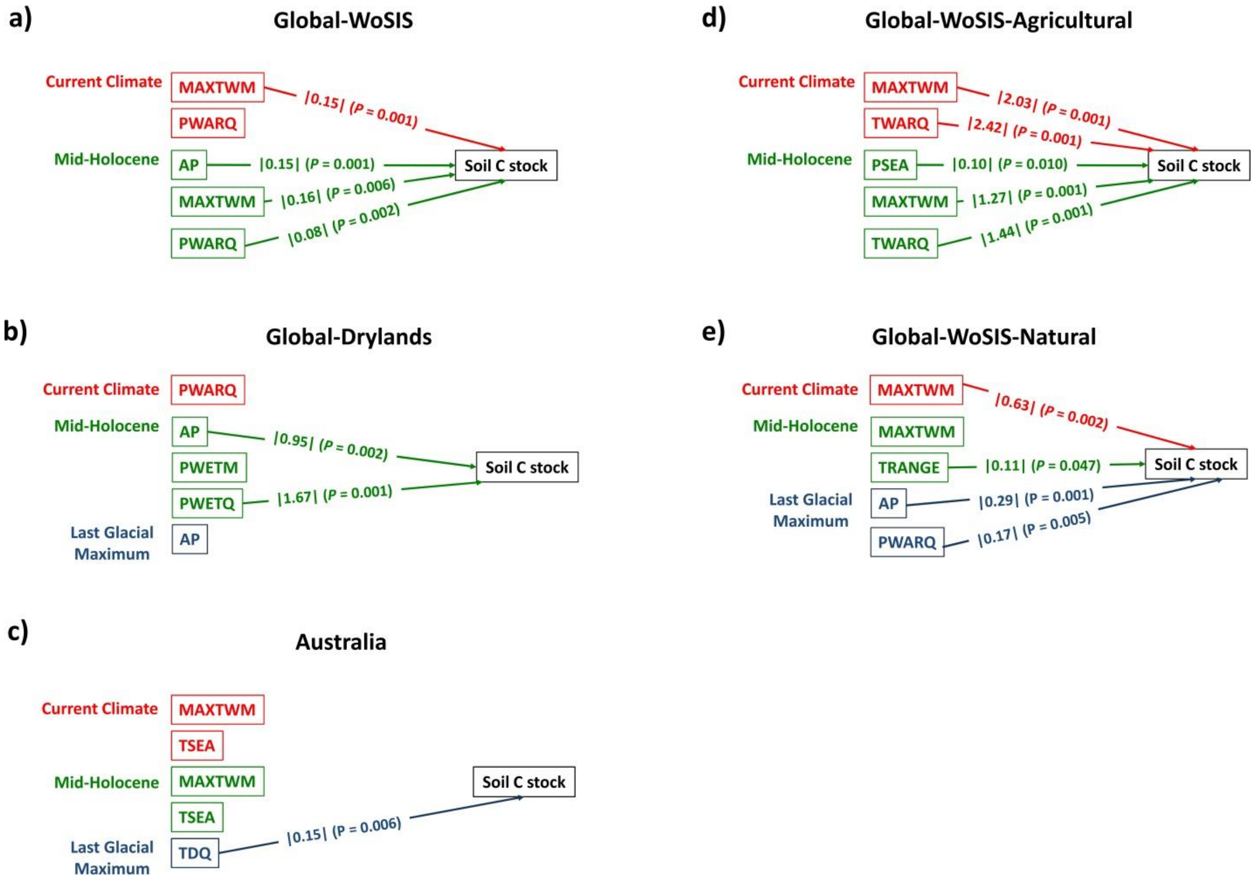

fig. S8. Structural equation modeling aiming to identify the relative influence of the main bioclimatic variables from current, mid-Holocene, and land maximum climate (as identified by random forest analyses) on soil C stocks. See table S5 for detailed information on all direct effects (significant and non-significant) from past and current climatic variables on soil C stocks and for correlations among exogenous variables in our models.

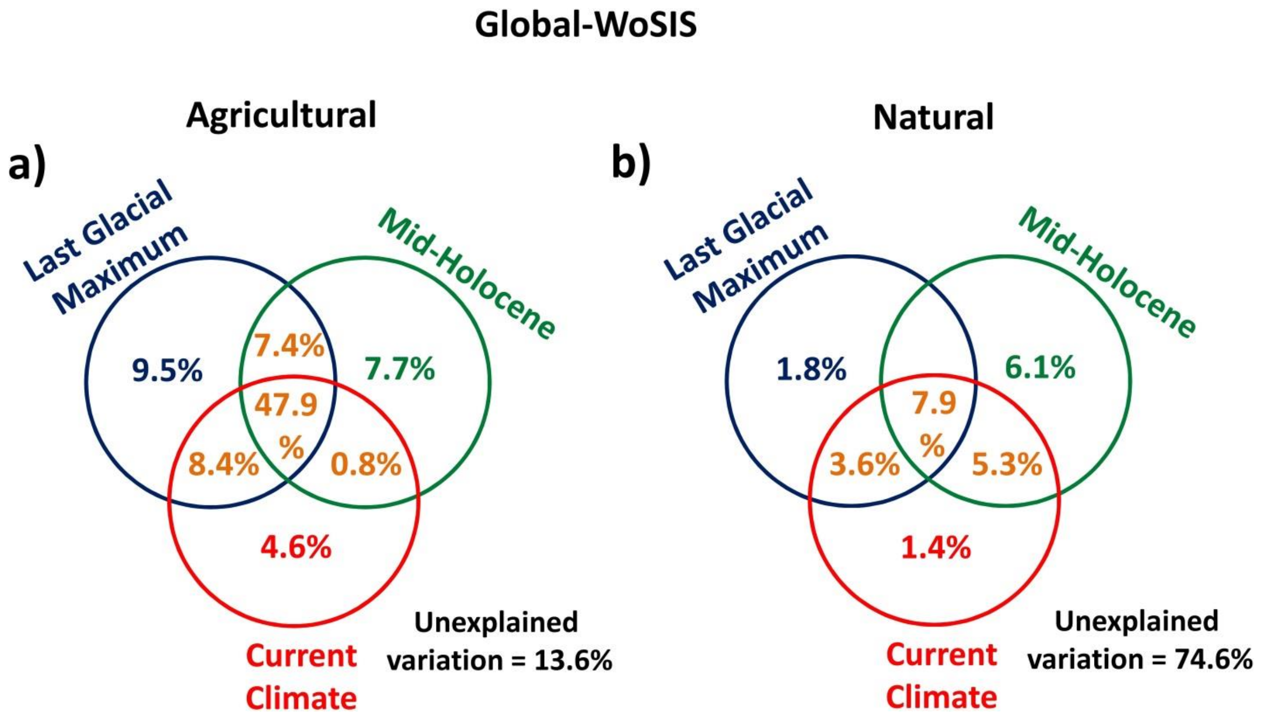

fig. S9. Relative contribution of paleo (mid-Holocene and Last Glacial Maximum) and current climate as drivers of the residuals of soil C stocks (from a multilinear regression with latitude and longitude as predictors of soil C stocks) in agricultural (*n* = 1167) and natural (*n* = 814) systems from the Global-WoSIS data set. Variation partitioning modelling aiming to identity the % variance of the residuals of soil C stocks explained by past and current climate variables for the identified agricultural and natural systems from the GLOBAL-WoSIS. Shared effects of these variable groups are indicated by the overlap of circles.The main goal of these analyses was to reduce the noise derived from spatial variables on soil C stocks. Thus, the residuals from these multi-linear regressions were not influenced by either latitude or longitude.
